# Adapted forest management to improve the potential for reindeer husbandry in Northern Sweden

**DOI:** 10.1007/s13280-023-01903-7

**Published:** 2023-07-31

**Authors:** Jeannette Eggers, Ulrika Roos, Torgny Lind, Per Sandström

**Affiliations:** Department of Forest Resource Management, Swedish University of Agricultural Sciences, SLU, 901 83 Umeå, Sweden

**Keywords:** Boreal forest, Forest management, *Rangifer tarandus*, Scenario analysis

## Abstract

In northern Sweden, improvements of grazing conditions are necessary for the continuation of traditional, natural pasture-based reindeer husbandry. Ground and tree lichen constitute the main fodder resource for reindeer during winter but have reached critically low levels. Using a forest decision support system, we prescribe adapted forest management to improve the preconditions for reindeer husbandry and compare outcomes with the continuation of current forest management. We found that adapted management increases the forest area with ground lichen habitat by 22% already within 15 years, while a continuation of current management would result in a further decrease in ground lichen. Tree lichen habitat can be retained and increased in all scenarios, which is important in a changing climate. Compared to a continuation of current practices, adapted management with significantly improved conditions for lichen resulted in a decrease in net revenues from wood production by 11–22%.

**Supplementary Information:**

The online version contains supplementary material available at 10.1007/s13280-023-01903-7.

## Introduction

Balancing overlapping and competing land uses is challenging in most ecosystems globally, in part due to conflicting interests, imbalanced power relations as well as lack of knowledge regarding the long-term consequences of different land management options. Holistic landscape approaches that include people and communities as part of the landscape can provide the scientific basis for policy choices regarding ecosystem management (Garedew et al. 2009; Svensson et al. 2012). To understand impacts and facilitate planning among land users, scenario analysis can be a valuable tool in search of balanced and sustainable solutions (Eggers et al. 2019, 2022). We exemplify the common dilemma of multiple and conflicting land users operating in the same area in the northern half of Sweden, where reindeer husbandry and forestry have divergent objectives reflected in their forest use. Here, forest owners’ primary objective is commonly wood production on their property, whereas reindeer herders are dependent on forests as a part of a pastoral landscape, where the main winter fodder resource for the reindeer is terrestrial and epiphytic lichen (Heggberget et al. 2002) (hereafter termed ground and tree lichen). The complexity of this dilemma is magnified by differing and competing views of what constitute landscape perspectives. In forestry, individual forest stands and property boundaries usually constitute the landscape in focus. In reindeer husbandry on the other hand, focus is on a much larger pastoral landscape that at times also spans several grazing seasons (Sandström 2015; Harnesk 2022). Furthermore, Roturier and Roué (2009) discuss the Sami word and concept *guohtun* that in addition to explaining the amount of grazing resources, also incorporates the distribution and accessibility of grazing resources. In this context, barriers caused by other land uses as well as snow conditions become important factors to consider.

Winter grazing and ground lichen in particular is recognized as the bottleneck resource in reindeer husbandry, while tree lichen is an especially important resource at times when snow conditions make the ground lichens inaccessible to the reindeer. Such winters, with difficult snow conditions, are becoming increasingly common under changing climate conditions, which further elevates the problem (Eira et al. 2013; Skarin et al. 2021; Rosqvist et al. 2022).

The pastoral reindeer husbandry system constitutes historical legacy, closely connected to the culture, tradition and well-being of the indigenous Sami people (Lundmark 2010). The Reindeer Husbandry Act (1971, p. 437) defines the exclusive rights for the Sami people to herd and graze their reindeer (*Rangifer tarandus*) on 55% of the Swedish land area, divided into 51 individual reindeer herding communities (RHCs). In this area, RHCs have the right to graze on all land regardless of ownership, and this grazing right is considered equal to ownership rights according to legal scholars (Hahn 2000; Brännström 2017). However, no areas are reserved exclusively for reindeer husbandry as other land uses always co-occur (Sandström 2015).

Industrial forestry started in northern Sweden during the 1850s (Östlund 1993; Widmark 2009), and has since then been transforming the forest landscape and changed the conditions for pastoral reindeer husbandry. One main objective of forest owners as well as forest policy has been to increase wood production. Forestry practice has changed from mainly uneven-aged forestry to even-aged forestry affecting almost all productive forests after the Second World War (Östlund et al. 1997; Lundmark 2010). Since then, the changed management has resulted in a continual densification of forests with higher growing stock, higher growth rates, increased harvest levels and larger areas of young and dense forests. The growing stock and harvest volumes have increased with more than 60% since the 1950s (SLU 2022). Forest regeneration has been intensified using soil scarification and planting. In combination with fertilization and plantation of Lodgepole pine (*Pinus contorta)*, this has led to a densification of forest stands, which is a major contributing factor to the ground lichen decline with 71% since the 1950 (Sandström et al. 2016; Tonteri et al. 2022; Horstkotte et al. 2023).

During the same period, tree lichen-abundant forests have also declined considerably as modern clear-cut forestry practices have become more widespread, causing significant shifts in age structure towards younger forests (Esseen 2019). As an example, tree lichen decline was estimated to 51% in a study area in the county of Norrbotten between 1926 and 2006 (Horstkotte et al. 2011). Furthermore, testimonies from reindeer herders state that both ground and tree lichen resources have reached a critical tipping point where traditional, natural pasture-based reindeer husbandry based on naturally occurring winter foods is severely threatened.

The pastoral reindeer husbandry system in Sweden includes migrations between and within seasonal grazing grounds. Similar to the pastoral system of the Sami people, an additional ca 20 indigenous groups practice reindeer husbandry across the Eurasian arctic, often overlapping with other land use forms such as forestry, mining, oil and energy exploration (Oskal et al. 2009). Of specific importance to the reindeer husbandry year are the spring migration to the calving grounds close to the summer grazing grounds, and the autumn migration back to wintering areas. The mountain RHCs migrate between the forestland and the mountains, while the forest RHCs migrate within the forestland. A functional wintering area for reindeer consists of a varied forest landscape that offers grazing opportunities at different weather and snow conditions (Roturier and Roué 2009; Harnesk 2022; Horstkotte et al. 2022). Besides the availability of lichen, important issues for reindeer herders include mobility through forests for both reindeer and herders, in particular along migration routes. The reindeer herders repulse Lodgepole pine plantations, as dense stands with low branches hinder the movements of both reindeer and herders, as well as reducing the occurrence of ground lichen (Horstkotte et al. 2023). In addition, intensive soil scarification can be a physical obstacle for the reindeer as well as destroying ground lichen areas (Roturier and Bergsten 2006; Svenska Samernas Riksförbund 2019).

The prerequisites for traditional, natural pasture-based reindeer husbandry are also threatened from other activities such as mining, wind power installations and other infrastructure extensions, in combination with climate change with altered weather and snow conditions (Sandström 2015; Fohringer et al. 2021; Skarin et al. 2021). Further stressors on the pastoral reindeer husbandry system are predators and tourism, and the combined effects of these cumulative pressures are occurring over large areas (Stoessel et al. 2022). However, forestry affects the largest area and directly the lichen resources. At the same time, forestry constitutes a land use activity Sami reindeer herders can influence and that could improve grazing conditions, if forestry practices are adapted to the needs of reindeer husbandry.

Together, private (SCA AB, Holmen Skog AB and several smaller companies) and state-owned (Sveaskog AB and the National Property Board) forest companies own and manage about half of the productive forests in the reindeer husbandry area (Sandström et al. 2016). Since 1923, some form of joint planning of forestry and reindeer husbandry has taken place through consultations (Skuncke 1955; Roos et al. 2022). Consultations in its present form have been legislated since 1990 (SFS 1979) and are mandatory for large forest owners (owning > 500 ha) on the year-round grazing grounds (SKFS 2015). The authorities recommend also including winter grazing grounds in the consultations. All larger private forest companies and the state-owned forests are certified by FSC, where consultation is mandatory also on winter grazing grounds (FSC 2020). At annually recurring consultations, each forest company presents their planned areas for final felling to the affected RHC. The consultations can also include regeneration measures, areas for fertilization, cleaning, thinning, planned forest roads and choice of tree species for regeneration. In separate reviews of the consultation procedures, Roos et al. (2022) and Widmark (2009) showed that the reindeer herders experience a lack of influence during consultations.

One way to improve the co-planning of forestry for timber production and reindeer husbandry would be to include the reindeer herders’ landscape perspective into the forest planning process, as suggested at least since 1954 by Skuncke (1955). As the reindeer move in the landscape, they have different needs in different times of the year. However, in practice today, consultation is only carried out on the level of individual forest stands based on forest ownership. As forestry is the financially stronger actor, they have defined the content and level of the consultations (Roos et al. 2022). One way forward could be to explicitly include aspects important for reindeer husbandry into the forest planning process at the company level. The forest companies use decision support systems (DSS) for determining the long-term planning of harvest levels on their forestland (Nilsson et al. 2013; Ulvdal et al. 2022). The DSS is used to optimize the forest management in terms of economic return from forestry and non-declining timber flows. Aspects related to reindeer husbandry are largely treated by including a so-called planning reserve, i.e., a requirement that the amount of harvestable forest volume must always exceed the planned harvest volume. This requirement is meant to account for the uncertainty that the forest companies face regarding the outcome of the consultations. However, the extent to which management practices are adapted more specifically to the needs of reindeer husbandry, in terms of timing of thinnings and thinning grade, as well as cleaning, is very limited.

In previous studies, Korosuo et al. (2014) and Miina et al. (2020) showed that a continuation of current management practices would lead to a further decrease in ground lichen habitat. Horstkotte et al. (2016) showed that net present value was reduced by 10–11% between current management practices and adapted management in lichen-rich forests, but did not evaluate the effects of management practices on land with the potential to re-establish ground lichen if management is adapted to promote lichen growth. Hence, there is an urgent need to develop and agree upon new and adjusted forest practices. In particular, there is a need to identify management strategies that improve the availability of ground lichen and balance this with maintaining high wood production.

The aim of this study is to define and compare alternatives of forest management practices in terms of the outcomes for wood production and conditions for reindeer husbandry. In particular, we aim to answer the following research questions:

- How does the continuation of current forest practices affect conditions for reindeer husbandry in terms of habitat for ground and tree lichen and mobility across the landscape?
- How can reindeer-adapted forest management improve conditions for reindeer husbandry?
- What are the effects of the different forest management practices, in terms of wood production, production of lichen habitats and economic output?

We will do this by defining and comparing three different forest management scenarios for a large forest landscape in northern Sweden. The scenarios included a reference scenario continuing current management practices, and two scenarios with forest management practices that were adapted to the needs of reindeer husbandry. These scenarios were simulated in a forest decision support system for a time horizon of 50 years.

## Materials and methods

### Study area

**Fig. 1:**
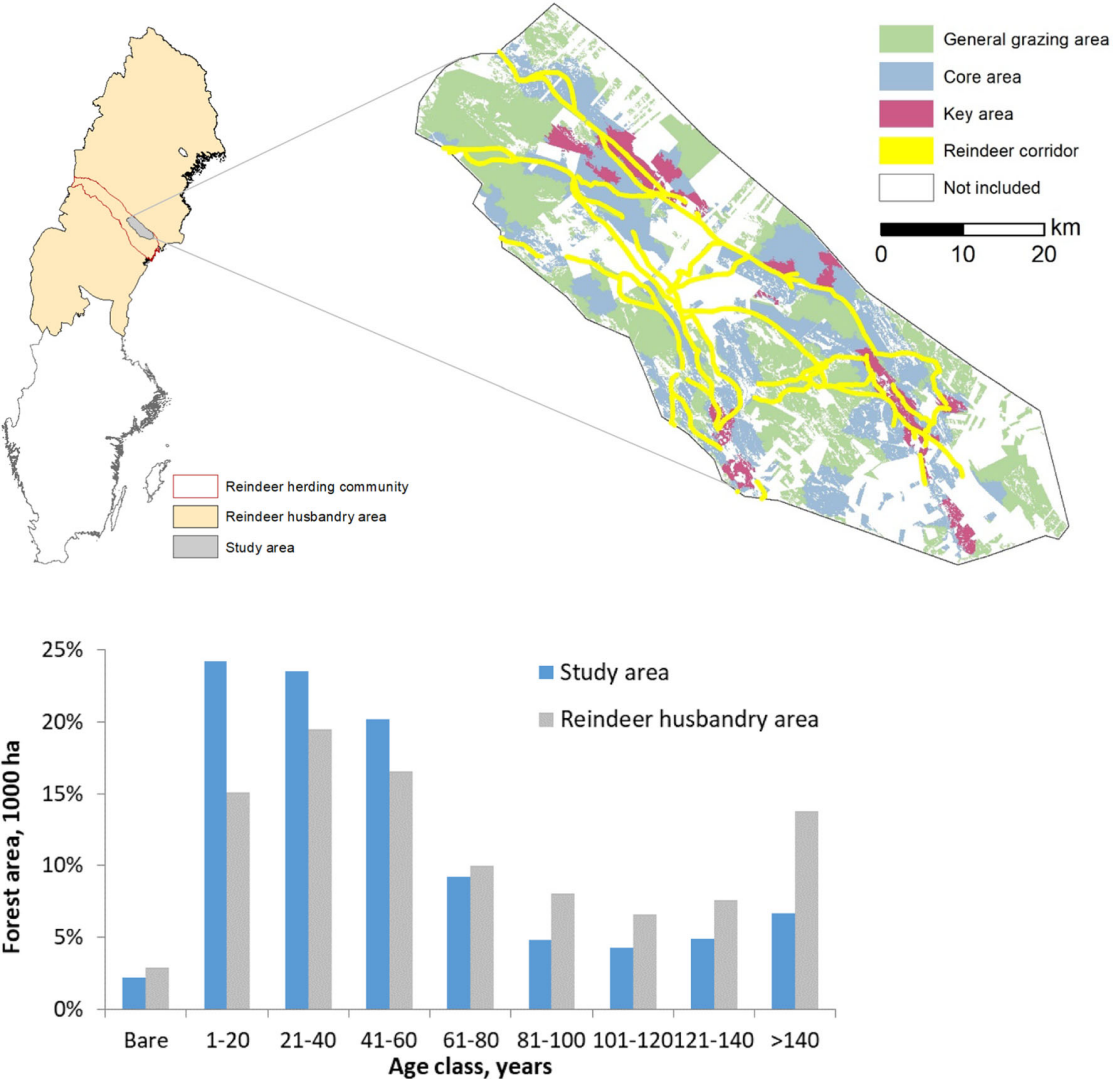
Overview of the study area: Its location within Sweden (top left), the division of the productive forest in the study area owned by the three forest companies divided into core, key and general browsing areas connected by reindeer corridors (top right), and the age-class distribution (lower panel). The age-class distribution includes the productive forests owned by the three forest companies in the study area, compared with the reindeer husbandry area. Area not included in the analysis includes forest owned by other owners, and other land uses

The study area is located in the County of Västerbotten in northern Sweden, on the winter grazing grounds of Vilhelmina Norra RHC (Fig. 1). The entire RHC covers 14 400 km^2^ where reindeer spend the snow-free seasons in western mountains and winters in the eastern coniferous boreal inland and coastal forests. The yearly movements of reindeer in the RHC can span more than 700 km from the west during summers, to the winter ranges in the east and back again via movement routes (reindeer corridors), which in part pass through our specific study area. The RHC is organized into different winter groups *(siida, sijdda, sïjte)* and our specific focus area is on the wintering lands of the winter group Vardofjällsgruppen covering 161 454 ha (Fig. 1). The forest companies SCA AB, Holmen Skog AB and Sveaskog own 117 050 ha of forest (72%) of the case study area, of which 115 421 ha is productive forestland, i.e., having a potential mean annual increment of more than 1 m^3^/ha/year. These companies provided stand-level information on management class, tree species distribution, site conditions, standing volume, age and basal area as input data for the analysis. Non-productive forestland is not managed, so we only considered the 115 421 ha of productive forests in the specific scenario analysis. The forest has a mean age of 52 years, with 70% of the area being younger than 61 years. The forest in the study area is thus younger compared to the average in the reindeer husbandry area (Fig. 1). The forest is dominated by Scots pine (*Pinus sylvestris*) with 65% of the growing stock, Norway spruce (*Picea abies)* 21%, birch (*Betula* spp.) and other broadleaved species 9% and Lodgepole pine (*Pinus contorta)* 5%.

In general, forests are managed for timber production using even-aged forestry, with stand-replacement cuttings followed by regeneration (mainly through planting after soil scarification), cleaning and one to three thinnings. Effective fire protection has eliminated fire as a disturbance factor. Till is the dominant soil type, with field layers including bilberry (*Vaccinum myrtillus*) and cowberry (*Vaccinum vitis-idea*). The forest floor layer is covered by leurocarpous mosses such as *Pleurozium schreberi* and *Hylocomium splendens* in mesic areas, while reindeer lichens (*Cladonia *spp.) dominate in drier parts. Especially during winters with difficult snow conditions, epiphytic tree lichens become especially important, including *Alectoria sarmentosa, Bryoria* ssp. and *Usnea* ssp.

Supported by our research team, Vilhelmina RHC has mapped and described habitat use and movement of reindeer across the landscape in their Reindeer Husbandry Plan (RHP), based on their expert knowledge and remote sensing techniques, further supported by GPS-data from reindeer (Fig. 1) (Sandström 2015). This division of grazing lands including key, core and general grazing areas is part of a dynamic and constantly updated process including new knowledge and changes in the landscape. Key areas contain the highest quality grazing lands, crucial for reindeer husbandry. Core areas are important areas regularly used for grazing, usually surrounding key areas. General grazing areas surround key and core areas and often delineate the entire lands used by a winter group. Grazing areas are connected through reindeer corridors both within seasonal grazing areas as well as for longer migrations between coastal forests and the mountains (Sandström 2015; Sametinget 2022). In our analysis, we used a standard width of 600 m for the reindeer corridors.

### Indicators for wood production and reindeer husbandry

To assess the impact of forest management on wood production and reindeer husbandry, we defined a number of indicators based on National Forest Inventory (NFI) data and earlier studies (Table 1).

**Table 1:** Indicators used to assess the impacts of forest management on reindeer husbandry and on wood production

Indicator	Definition
Forest area with potential ground lichen habitat	Area of pine-dominated forest, site index 12–20, dry or mesic soil, basal area < 18 m^2^/ha
Forest area with potential tree lichen habitat	Forest area with age > 60 years and a closure index ≥ 0.6
Forest area dominated by Lodgepole pine	Lodgepole pine has largest basal area or stem number among all species present in the stand
Density of forest in reindeer corridors	Basal area of trees within reindeer corridors (m^2^/ha)
Harvest volume	Volume extracted from the forest, divided into sawlogs and pulpwood (m^3^ub)
Net revenues from wood production	Gross revenue from timber and pulpwood minus costs for harvesting and silvicultural operations (EUR)
Annual area of thinnings, selections fellings and final fellings	Area (ha)
Net annual increment	Annual growth in tree volume, excluding natural mortality (m^3^ob)
Growing stock	Tree stem volume above the felling cut. Includes bark and top of the tree, but not branches (m^3^ob)

According to NFI data (SLU 2022), the majority of lichen-abundant/lichen-moderate plots in the reindeer husbandry area (about 90%) were situated in Scots pine-dominated forests with a site index between 12 and 19 (Supplementary material Table S1), and on dry and mesic sites (98%) (Supplementary material Table S2). However, since such class boundaries are not exact we also include pine forest with a site index of 20 as our indicator to have the potential to maintain/colonize/re-colonize ground lichens. A basal area of 15 m^2^/ha has been reported as optimal for lichen growth (Jonsson Čabrajič et al. 2010), abundance (Sandström et al. (2016) and decline (Horstkotte and Moen 2019). While ground lichen occur also in forests with higher basal areas, lichen occurrence declines significantly in forests with a basal area over 20 m^2^/ha (Sandström et al. 2016). Therefore we use pine-dominated forests, with a basal area below 18 m^2^/ha, on dry and mesic sites as an indicator for lichen habitat.

In forest stands older than 63 years, the presence of tree lichen becomes more common (Horstkotte et al. 2011; Horstkotte and Djupström 2021). As tree lichen disappear completely after clear-cuts, selective fellings and longer rotations are directly beneficial to increase potential tree lichen presence in the landscape (Rikkonen et al. 2023). At the same time, open stands with canopy closures below 70% often lead to a decline in tree lichen occurrence (Boudreault et al. 2013). Below this threshold, there is a risk for the lichen to dry out or to blow away by wind. Since simulations of canopy closure was not possible in the forest decision support system used in our study, we used a closure index based on the ratio between the actual forest volume, compared to the volume that would be optimal to fully use the wood production potential of the site. Based on a comparison of this closure index with canopy closure using NFI data, we chose 0.6 as threshold for the closure index.

Lodgepole pine-dominated stands are denser than domestic conifer stands (Bäcklund et al. 2018). Ground lichen cover was found to be lower in Lodgepole pine stands compared to domestic pine (Bäcklund et al. 2015) and the needle litter cover was more than three times greater (Nilsson et al. 2008). Also, Lodgepole pine-dominated forest aggravates the work for the reindeer herders, since the Lodgepole pine plantations are hard to pass through both for reindeer and herders (Svenska Samernas Riksförbund 2019). Therefore, the forest policy of the National Confederation of Swedish Sami states a zero tolerance against the planting of Lodgepole pine, and demands transformation of existing plantations to domestic species (Svenska Samernas Riksförbund 2019).

### Scenario analyses and modelling framework

Scenario analysis is a useful method for exploring plausible futures (Bengston et al. 2012). Long-term scenario analyses are a common way to analyze and compare outcomes of forest management practices (Peterson et al. 2003). In this study, we defined three scenarios with different management practices based on consultation with representatives for the forest companies and the RHCs. The consultations even included in-depth discussions of the chosen indicators as well as preliminary results, allowing us to adapt the simulations according to the comments we received.

The scenarios were:

- Reference—Current forest management practices as defined by the forest companies.
- Ground lichen—Forest management practices with the objective to increase the area with ground lichen habitat and mobility of reindeer.
- Ground and tree lichen—Forest management practices with the objective to increase the area with ground and tree lichen habitat and mobility of reindeer.

The development and wood production of the productive forest in the study area for the three scenarios were simulated 50 years into the future, using the forest decision support system (DSS) for long-term analysis and planning of the forest landscape Heureka PlanWise (version 2.18.3.0) (Lämås et al. 2023). A simulation period of 50 years was chosen because it is the next decades that are decisive regarding the future of traditional, natural pasture-based reindeer husbandry, and because uncertainties regarding the tree layer development increase with longer time periods.

We simulated the development of the tree layer in 5-year time increments using a large set of empirical models simulating growth, mortality and ingrowth. Expected impacts of climate change on forest growth were accounted for by adjusting the empirical growth functions using the BIOMASS process-based vegetation model (McMurtrie et al. 1990) for the RCP4.5 radiative forcing scenario (Thomson et al. 2011) as modelled with the MPI-ESM model (Giorgetta et al. 2013). To account for the expected increased risk of disturbances due to climate change (Venäläinen et al. 2020), which is not covered by the vegetation model, we increased the sapling damage factors in young forest, and natural mortality in established forests, by 20% in the simulations. Models calculating cost for forest management and revenues from wood products is also included in the DSS. For individual trees, height growth in young stands is simulated (mean height < 7) (Fahlvik and Nyström 2006) basal area growth for established stands (mean height ≥ 7 m) (Fahlvik et al. 2014), and mortality (Elfving 2014). Heureka PlanWise also includes models simulating the effects of treatments such as pre-commercial thinning, thinning, final felling, regeneration methods, fertilization and climate change.

The productive forest’s stands were grouped by forest type, i.e., groups of stands with similar properties. The grouping differed between scenarios, both in terms of number and properties of groups. Each forest type was linked to one or more forest management strategies. Management strategies can differ in management regime (unmanaged, uneven-aged or even-aged management), or in how different management actions (such as regeneration, cleaning and thinnings) are performed. PlanWise simulates treatment schedules for each stand and management strategy, and finds the optimal combination of treatment schedules in the landscape using linear programming. In the optimization, a user-defined goal is maximized or minimized with considerations to constraints at stand, forest type and forest level. PlanWise can report results for many indicators such as tree species distribution, harvest volume distributed on assortments, growing stock, growth, mortality, biomass content, carbon in trees and soil, area of management activities as thinning and final felling, and costs and revenues.

#### Management strategies

The management strategies applied in the three scenarios were based on written and oral consultations with representatives for the three forest companies owning the majority of forestland in the case study area, and reindeer herders using the forests in the area for winter grazing. The consultations took place during the spring of 2021.

**Fig. 2:**
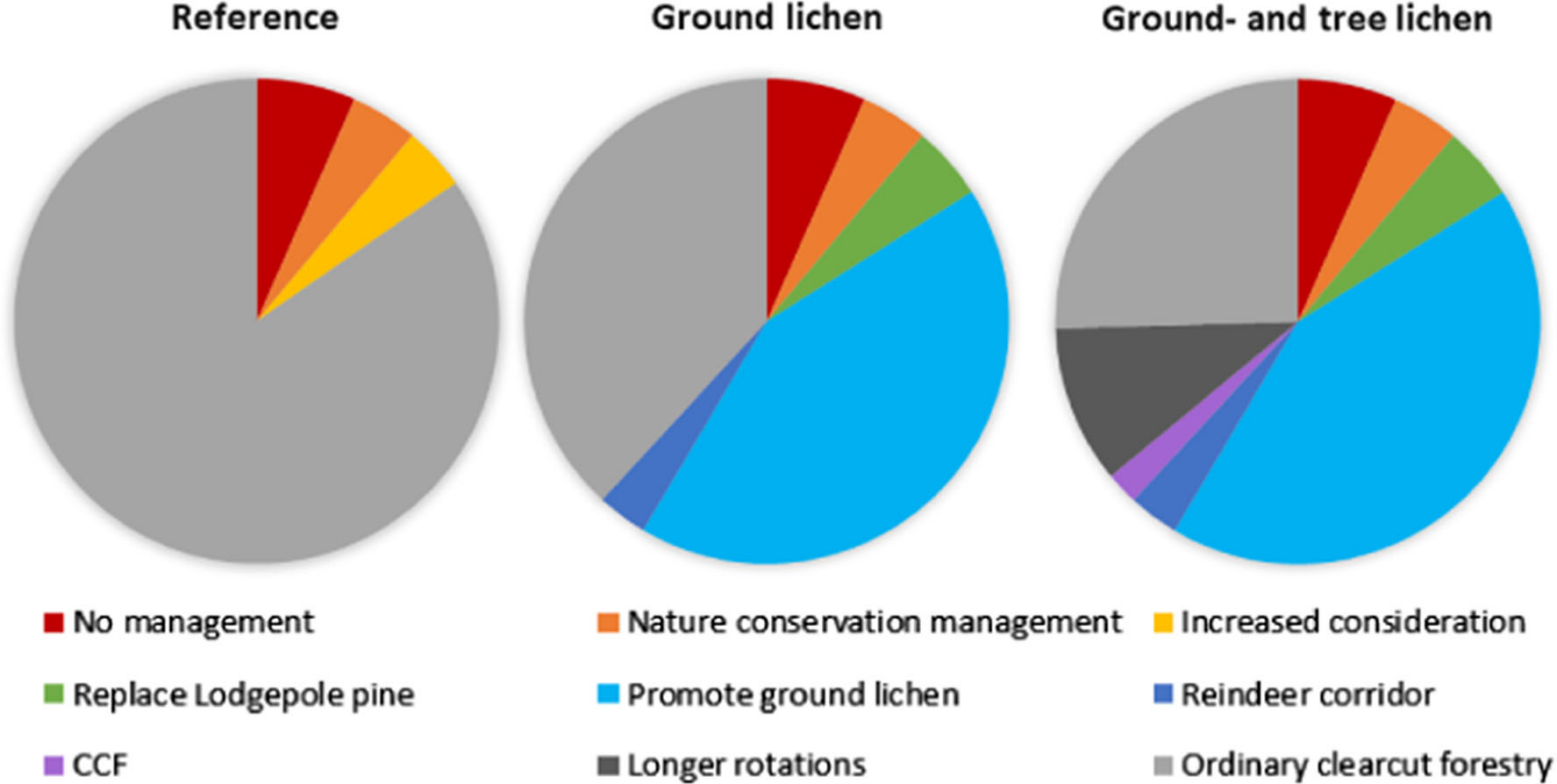
Distribution of forest area assigned to different management strategies in the three scenarios

The forest companies delivered information on which part of the forest is set-aside for nature conservation, with or without management, and which part of the forest is assigned for selective fellings. These forest areas were managed in the same way in all three scenarios (Table 2). The remaining forest area was managed differently in each scenario (Fig. 2), as described in detail in the next sections. In all scenarios, an interest rate of 2.5% was used for the calculation of the net present value.

**Table 2:** Management strategies common in all three scenarios

Forest type	Forest area (ha)	Share of total area, %	Management
Forest set-aside for nature conservation, without management	7625	6.6	No management
Forest set-aside for nature conservation, with management	4690	4.1	Management aiming to improve nature values: thinnings that remove conifers and thus improve growing conditions for broadleaves
Forest assigned for continuous cover forestry	491	0.4	Selective fellings, implemented as thinnings from above

##### Reference scenario

The forest that was not set-aside for nature conservation was managed with even-aged forestry (Table 3). The state-owned forest company Sveaskog stated they are phasing out Lodgepole pine in the area, thus their Lodgepole pine forests are regenerated with planted Scots pine. For the two other forest companies, planting Lodgepole pine is an option also in the future. Furthermore, fertilization is an option for all productive forest outside key and core grazing areas and has the vegetation type bilberry. No specific management consideration was made for reindeer corridors.

**Table 3:** Management strategies in the Reference scenario

Forest type	Forest area (ha)	Share of total area, %	Management
Forest assigned to management with increased consideration for other values	4896	4.2	Prolonged rotation period, natural regeneration, 40% broadleaves left in cleanings and thinnings
Other forest	97 719	84.7	Business-as-usual clear-cut forestry. Regeneration with plantation of Scots pine or Norway spruce, pre-commercial thinnings, up to 3 thinnings, final felling within 30 years of reaching the minimum final felling age. Regeneration with Lodgepole pine possible for SCA and Holmen, in areas currently dominated by Lodgepole pine, or situated outside key and core areas for reindeer husbandry and with bilberry vegetation type. Final felling of Lodgepole pine at 55–60 years of age and regeneration with Scots pine (Sveaskog). Fertilization possible outside key and core areas for reindeer husbandry, in forests with bilberry vegetation type

For all forest types except forest dominated by Lodgepole pine, 12% of the forest area was left as retention patches after final felling. In addition, three high stumps were left per ha in thinnings and final felling, and 10 retention trees in final fellings.

##### Ground lichen scenario

The forest stands were grouped into forest types, and these types were assigned different management strategies (Table 4, Fig. 2). Lodgepole pine was removed and replaced with Scots pine. In reindeer corridors, key and core grazing areas Logdepole pine stands were harvested at 30 years of age, in other areas, at 55–60 years of age.

In forests with potential for occurrence or re-establishment of ground lichen, i.e., in pine-dominated forests with a site index (SI) of 12–20 on dry or mesic sites, a management strategy was applied that maintained a low basal area throughout the rotation. On the least fertile sites, natural regeneration was applied, while on higher fertility sites, regeneration was through plantation following a careful soil scarification. No soil scarification was used with natural regeneration, to avoid negative effects of site preparation on ground lichens (Eriksson and Raunistola 1990; Roturier and Bergsten 2006). Cleanings and thinnings were adapted to allow for earlier and more intensive practices in terms of cut stems/volume, but in accordance with the legal regulations for forest management (SKFS 2015) (for details, see Table 4). We assumed an extra cost of 15%, added to the hourly harvester and forwarder cost, for piling up harvest residues in thinnings and final fellings so that they do not cover the ground lichen.

**Table 4:** Management strategies applied in the different forest types in the Ground lichen scenario

Forest type	Forest area (ha)	Share of total area, %	Management
Forest dominated by Lodgepole pine in reindeer corridor, key or core grazing area	616	0.5	Remove and replace with Scots pine when Lodgepole pine is 30 years old, maintain low basal area in Scots pine forest (cleaning to 1200 stems, thinning curve ratio 0.65)
Forest dominated by Lodgepole pine, SI ≤ 20 on dry and mesic site	959	0.8	Remove and replace with Scots pine when Lodgepole pine is 55–60 years old, maintain low basal area in Scots pine forest (planting, cleaning to 1200 stems, thinning curve ratio 0.65)
Other forest dominated by Lodgepole pine	4128	3.6	Remove and replace with Scots pine when Lodgepole pine is 55–60 years old, manage Scots pine with standard clearcut forestry
Pine-dominated forest on dry and mesic sites, SI 12–16	3486	3.0	Maintain low basal area (natural regeneration, cleaning to 800–1000 stems, lower and upper thinning curve limits reduced by 35%), minimum felling age increased with 30%, pile up harvest residues
Pine-dominated forest on dry and mesic sites, SI 17–18	14 163	12.3	Maintain low basal area (plantation (1000 plants/ha), cleaning to 800–1000 stems, thinning curve ratio 0.65), minimum felling age increased with 20%, pile up harvest residues
Pine-dominated forest on dry and mesic sites, SI 19–20	31 372	27.2	Maintain low basal area (plantation (1000 plants/ha), cleaning to 1200 stems, up to 4 thinnings, thinning curve ratio 0.65), minimum felling age increased with 20%, pile up harvest residues
Reindeer corridors	3880	3.4	1200 stems/ha in planting and after cleaning, lower basal area by intensive thinnings (thinning curve ratio 0.8, minimum final felling age increased by 10%), pile up harvest residues
Other forest	44 012	38.1	Standard clearcut forestry. Regeneration with plantation of Scots pine or Norway spruce, pre-commercial thinnings, up to 3 thinnings, final felling within 30 years of reaching the minimum final felling age. No plantation of Lodgepole pine and no fertilization

In the reindeer corridors, management aimed to keep an open forest through earlier and more intensive cleanings and thinnings, to allow for reindeer to move through the landscape and for the herders to monitor the reindeer during migration.

No forest fertilization was applied in this scenario. For all forest types except forest dominated by Lodgepole pine and forest set-aside for nature conservation, 10% of the forest area was left as retention patches at final felling. In addition, three high stumps were left per ha in thinnings and final felling, and 10 retention trees in final fellings.

##### Ground and tree lichen scenario

The Ground and tree lichen scenario used the same forest management strategies as the Ground lichen scenario for all forest types except for other forest (last row in Table 4), and retention settings. Because local dispersal of tree lichen is limited in young stands (Dettki et al. 2000), the retention and creation of old, tree lichen-rich forest patches has been suggested to be an efficient strategy to promote tree lichen abundance. To support the dispersal of tree lichen, the size of retention patches left at final felling was increased to 20% of the forest stand in stands larger than 9 ha (Esseen 2019), as larger retention patches are more likely to retain tree lichen after the surrounding forest has been cut. Management of other forest was adjusted by adding continuous cover forestry as a management strategy for uneven-aged spruce forest on 2.2% (2496 ha), and prolonging the minimum final felling age with 30% for even-aged spruce forest on 10.5% (12 148 ha) of the study area.

#### Optimization

In the Reference scenario, net present value was maximized with a 2.5% interest rate, with the following constraints: Final felling volume was not allowed to decrease with more than 2% between consecutive periods, aiming for even final felling volumes. At the same time, final felling volume was not allowed to increase with more than 10% between consecutive periods. For Sveaskog, we applied a volume reserve of 2.8 times the final felling volume. That is, for each m^3^ subject to final felling, there must be 2.8 times that volume in stands available for final felling, i.e., above the minimum final felling age. For the other owners, we applied an area reserve of 1.15: for each hectare subject to final felling, there must be 1.15 ha of area available for final felling. For the forest owned by SCA or Holmen, the annual area regenerated with Lodgepole pine was required to be between 1 and 3%. According to official statistics (Swedish Forest Agency 2022), between 0.04 and 0.3% of the productive forest area in northern Sweden has been fertilized annually during the last 10 years. We assumed that this will continue, resulting in the restriction that between 0.2 and 1.5% of the forest area would be allowed to be fertilized per 5-year period.

In the Ground lichen scenario, the optimization maximized the area with ground lichen habitat over time, i.e., the average over the planning horizon of 50 years. The ground lichen habitat was not allowed to decrease with more than 1% between consecutive periods. As in the Reference scenario, final felling volume was not allowed to decrease with more than 2%, or increase with more than 10%, between consecutive periods.

In the Ground and tree lichen scenario, we used the same optimization model as the Ground lichen scenario; with the addition that potential tree lichen habitat was not allowed to decrease over time with more than 1% between consecutive periods.

## Results

**Fig. 3:**
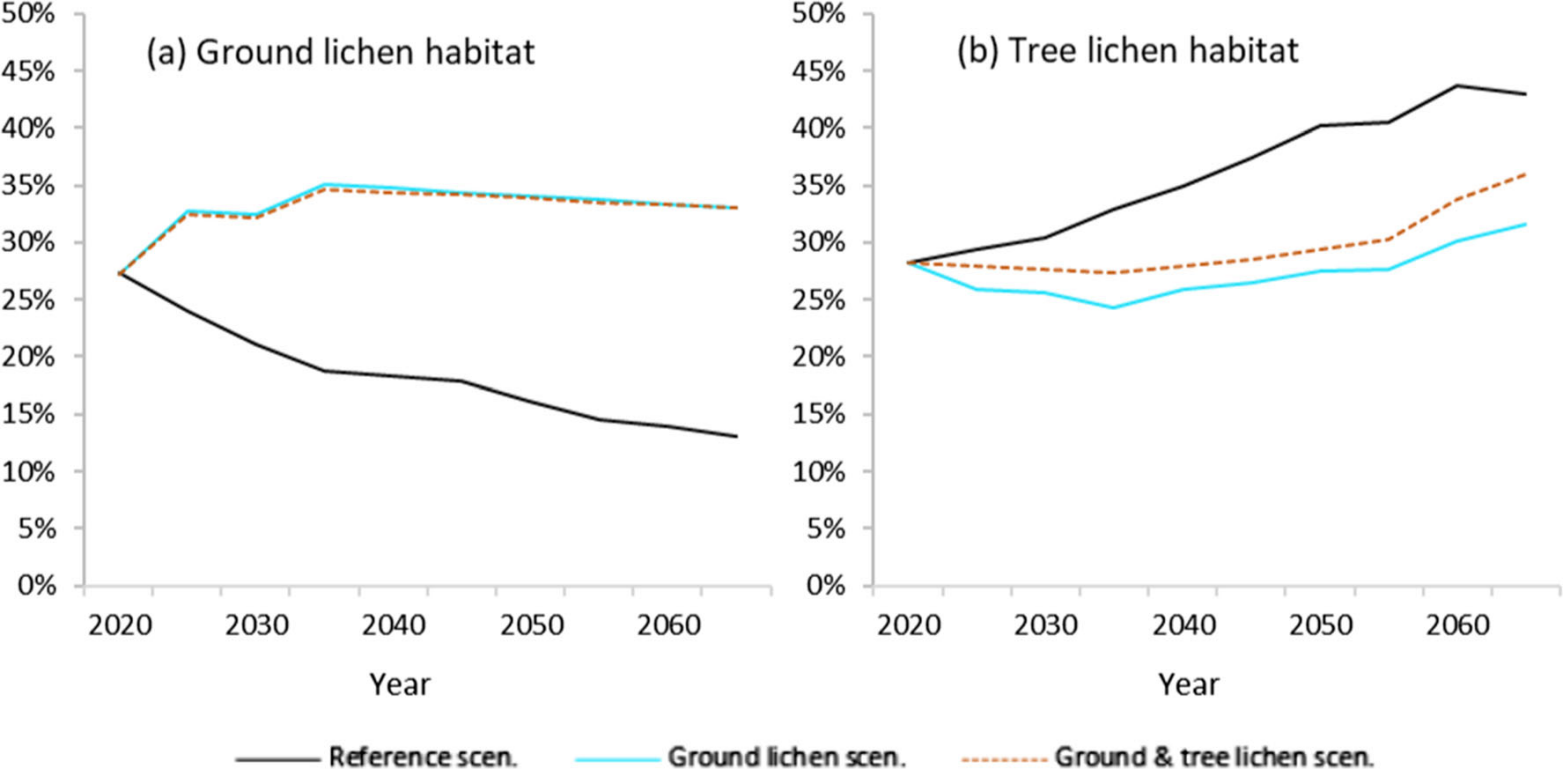
Development of the proportion of forest area with potential for ground lichen (**a**) and for tree lichen (**b**)

In the Reference scenario, the area with ground lichen habitat decreased steadily throughout the 50 year study period (2020–2070) (Fig. 3a), continuing the declining trend observed for the past 70 years (Sandström et al. 2016). The proportion of forests with ground lichen habitat decreased with 50%, from the present 27% of ground lichen habitat to only 13% at the end of the study period. On the other hand, the area with ground lichen habitat increased with 22% (from 27 to 35%) already during the first 15 years for the two lichen scenarios, and stabilized thereafter (Fig. 3b). Hence, the difference in the outcome for ground lichen habitat when comparing the Ground lichen scenario and the Reference scenario in 2035 shows 46% (35% vs 19%) more ground lichen habitat and at the end of the study period the difference was 60% more ground lichen habitat (33% vs 13%). For comparison, the forest area with adapted management for promoting ground lichen in both lichen scenarios was 49 000 ha, or 42% of the total productive forest area. Both lichen scenarios thus resulted in around 80% ground lichen habitat of the area managed for ground lichen. It is worth noting that results on the forest condition, including basal area, are reported for the middle of the 5-year period, before any management actions are performed. This can lead to conditions for the ground lichen indicator in terms of basal area not being met temporarily, for one 5-year period at a time.

The area with tree lichen habitat increased in the Reference scenario. In the Ground lichen scenario, tree lichen habitat decreased during the first 15 years, before starting to increase to levels slightly above the initial situation in the end of the study period (Fig. 3b). In the Ground and tree lichen scenario, tree lichen habitat remained stable for most of the study period, with an increase during the last 15 years. The mean age of forest classified as tree lichen habitat was 106 years in the beginning of the study period. The age remained stable initially and increased slightly to 110 years in both lichen scenarios, but decreased to below 100 years in the Reference scenario.

**Fig. 4:**
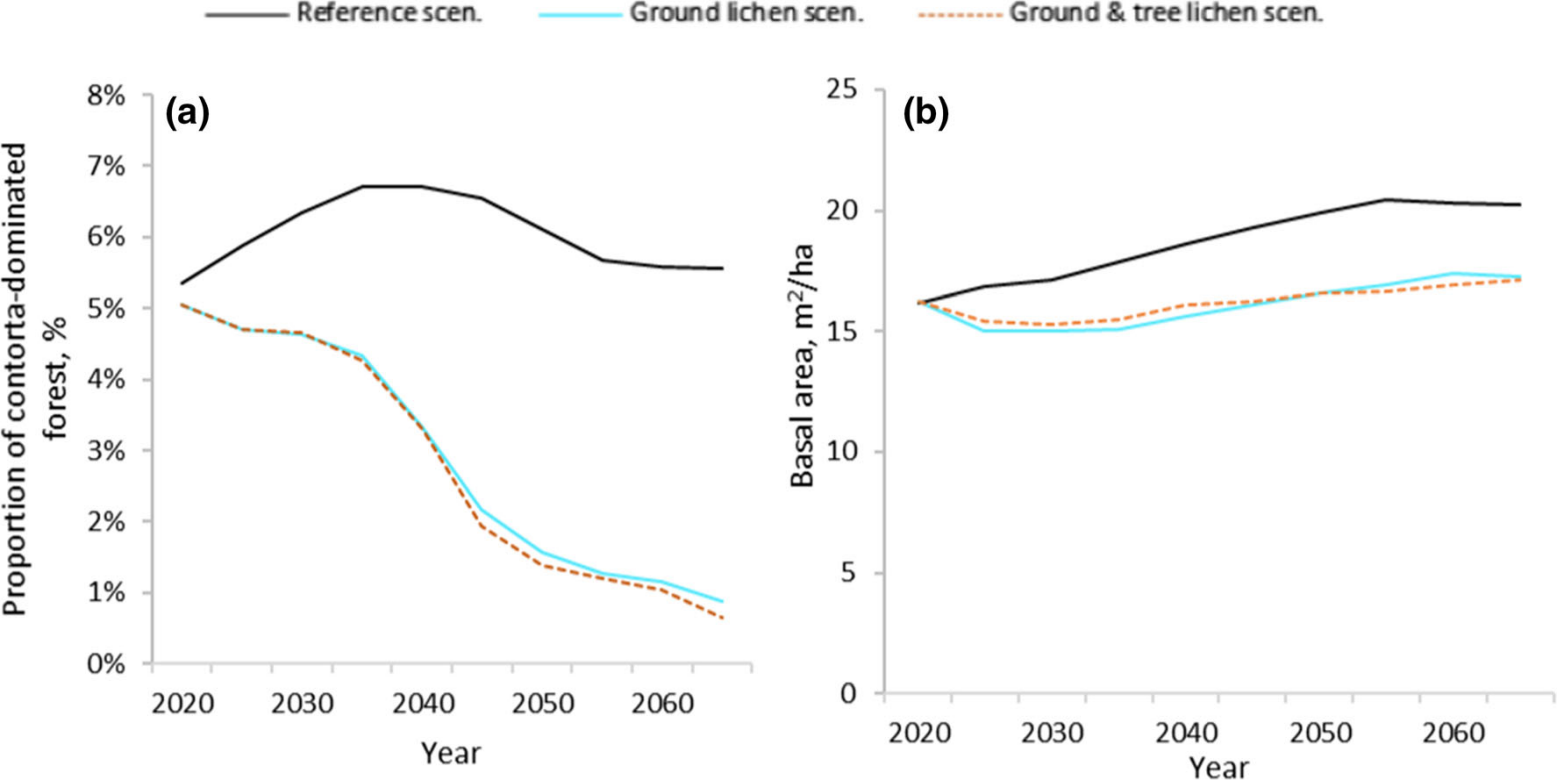
Development of the proportion of forest area dominated by Lodgepole pine over time (**a**), and average basal area of forest in reindeer corridors (**b**)

Lodgepole pine stands constitute 5% of the forest area in the beginning of the study period (Fig. 4a). The forest area of Lodgepole pine stands increased during the first 20 years in the Reference scenario, before returning to current levels. In both lichen scenarios, Lodgepole pine stands decreased steadily, down to less than 1% of the forest area after 50 years.

Average basal area in reindeer corridors was consistently lower, ranging between 15 and 17.5 m^2^/ha, in the lichen scenarios, compared to the Reference scenario in which the average basal area increased over time, to more than 20 m^2^/ha (Fig. 4b).

**Fig. 5:**
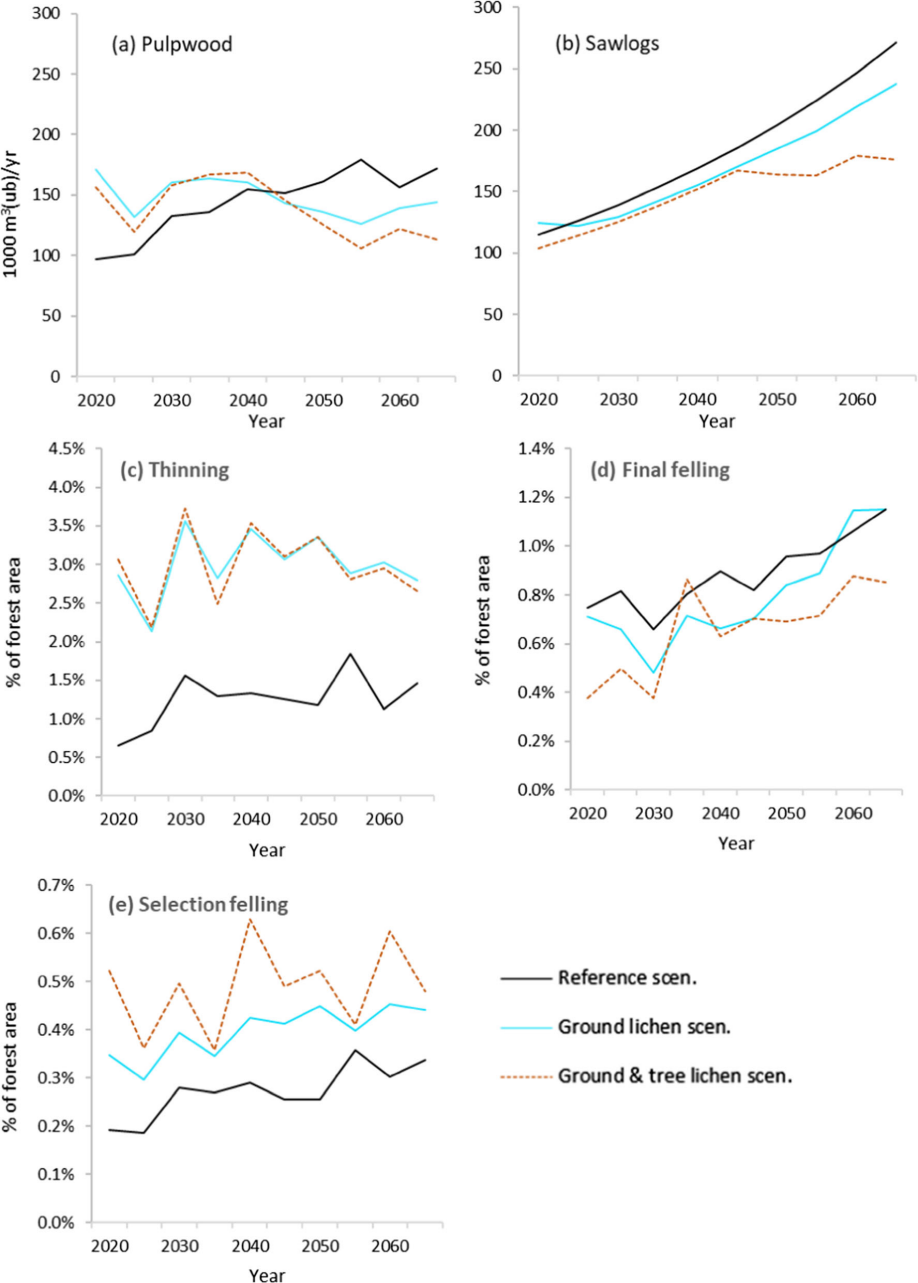
Development of annual harvested volume over time, distributed into **a** pulpwood and **b** sawlogs, and forest area annually subjected to thinning (**c**), final felling (**d**) and selection felling (**e**) in the three scenarios

In the Reference scenario, the annual harvested volume of both pulpwood and sawlogs increased over time (Fig. 5a, b). Both lichen scenarios resulted in higher harvest of pulpwood volumes during the first 25 years, due to larger areas of and harder thinnings to promote ground lichen, and lower pulpwood volumes afterwards compared to the Reference scenario. On average over the study period, pulpwood harvest was highest in the Ground lichen scenario (148 000 m^3^ year^−1^), lowest in the Ground and tree lichen scenario (138 000 m^3^ year^−1^) and in between for the Reference scenario (144 000 m^3^ year^−1^). The harvest of pulpwood in the Reference scenario was markedly lower than in the lichen scenarios during the first half of the study period and higher during the second half. Volumes of sawlog harvest increased throughout the study period in all scenarios. From 2045 and onward, sawlog volumes stabilized in the Ground and tree lichen scenario, while it continued to increase in the Reference and Ground lichen scenarios. In the Ground lichen scenario, sawlog volumes were higher than in the Reference scenario during the first 10 years and lower during the remainder of the study period. The lower harvest volumes in the Tree and ground lichen scenario in the second half of the study period, compared to the two other scenarios, can be explained by the longer rotation periods applied in that scenario to promote tree lichen.

The annual area thinned was more than twice as high for the lichen scenarios compared to the Reference scenario throughout the study period (Fig. 5c). Consequently, this resulted in higher volumes of harvested pulpwood (Fig. 5a). The final felling area for the Reference and Ground lichen scenario followed similar trajectories, and ended up identical at the end of the study period. For the Ground and tree lichen scenario, the final felling area was lower and fluctuated more. The annual area of selection fellings was about twice as high in the Ground and tree lichen scenario while the Ground lichen scenario was about 50% higher than the Reference scenario throughout the study period.

**Fig. 6:**
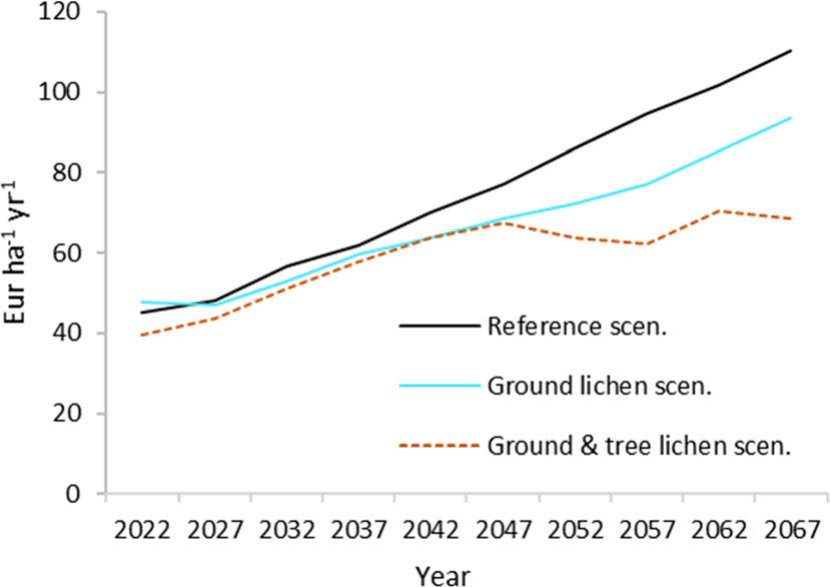
Development of net revenues from wood production over time. 1 EUR = 10 SEK

Net revenues from wood production increased over time in all three scenarios (Fig. 6). The increase ranged from 74% in the Ground and tree lichen scenario, to 145% in the Reference scenario. Net revenues in the Ground lichen scenario were slightly higher compared to the Reference scenario during the first 5 years and very similar during the first 25 years. In the Ground and tree lichen scenarios, net revenues increased during the first 25 year before stabilizing. On average over the 50-year study period, net revenues were 11% lower in the Ground lichen scenario, and 22% lower in the Ground and tree lichen scenario compared to the Reference scenario.

Net present value (NPV) was highest in the Reference scenario, 3305 Eur ha^−1^. In the Ground lichen and Ground and tree lichen scenarios, NPV was 10% (2969 Eur ha^−1^) and 13% (2880 Eur ha^−1^) lower compared to the Reference scenario, respectively. The discount rate was 2.5% and we assumed 1 EUR = 10 SEK.

Net annual increment (NAI) increased during the first 15 years in all three scenarios (see Fig. S1 in Supplementary material), from 3.8 to 4.2 m^3^ha^−1^ year^−1^ in the lichen scenarios, and 4.5 m^3^ha^−1^ year^−1^ in the Reference scenario. After 15 years, the NAI remained at same level in both lichen scenarios, while it continued to increase for another 20 years in the Reference scenario, to 4.8 m^3^ha^−1^ year^−1^. Growing stock increased in all scenarios as NAI exceeded the harvest level. The increase in growing stock was most pronounced in the Reference scenario (60% increase within the 50 year study period), and lowest in the Ground lichen scenario (43% increase).

## Discussion

We found that continued ‘business as usual’ forest management (the Reference scenario) would extend the past 70-years of decline of ground lichen habitat as our results for the Reference scenario show an additional 50% decline during the next 50 years. This represents an alarming trend, as today’s amount of lichen habitat already is recognized as critically low (unified statement of hundreds of reindeer herders, and explicitly stated by local reindeer herders in our study area). Such continued negative effects on the lichen resources thus severely threaten RHCs possibility to carry out traditional, natural pasture-based reindeer husbandry. By applying management strategies aiming to increase lichen habitat, the area with ground lichen habitat increased with more than 20% compared with today. In the lichen scenarios, around 80% of the area managed for ground lichen fulfilled our defined habitat requirements for ground lichen, which is twice as much compared to the Reference scenario. After 50 years, the amount of ground lichen habitat would be 2.5 times higher in the lichen scenarios, compared to the Reference scenario.

The area of tree lichen habitat increased over time in all scenarios, apart from a slight decrease during the first decades in the Ground lichen scenario. The tree lichen habitat increased most in the Reference scenario, mostly due to the increase of relatively dense forests older than 60 years. While tree lichen can occur in forests younger than 60 years, it takes time before the tree lichen are abundant in the stand. This dynamic cannot be captured in the tree lichen indicator we applied. However, the majority of the forest classified as tree lichen habitat is considerably older than 60 years. In the Reference scenario, the mean age of the forest classified as tree lichen habitat decreased over time, while it increased slightly in the lichen scenarios. This means that although the area of tree lichen habitat increased most in the Reference scenario, the abundance of tree lichen does not necessarily follow the same trend. In the lichen scenarios, more than 40% of the forest area is managed to promote ground lichen habitat, hence maintaining a low basal area. Such open forests can discourage the development of tree lichen habitat requiring more closed canopies (Boudreault et al. 2013). This result illustrates the importance to adapt management based on the particular goals and conditions at each site. It is difficult to focus forest management on both ground and tree lichen within the same stand. Our results point at a clear dividing point to focus ground lichen considerations on pine forests younger than 80 years, with a site index up to 20 on dry and mesic soils and focus on tree lichen in other forests, such as older mixed species stands. Under a changing climate, tree lichen is becoming increasingly important. How this affects the prioritization between ground and tree lichen needs to be investigated in future studies.

In the Ground and tree lichen scenario, we promote tree lichen by managing spruce-dominated forests with continuous cover forestry (2.2% of the forest area) or prolonged rotations (10.5% of the forest area). This leads to a larger increase of tree lichen habitat compared to the Ground lichen scenario, while remaining under the levels of tree lichen habitat reached in the Reference scenario. That is, the management adaptation to promote tree lichen in the Ground and tree lichen scenario did not fully compensate for the potential loss in tree lichen habitat caused by promoting ground lichen. However, the management measure to increase retention patches to 20% of the forest stand in the Ground and tree lichen scenario is likely to result in maintaining more tree lichens in these patches, compared to the smaller patch size of 10% in the other scenarios, in which tree lichen occurrence decreases during the first years after final felling due to wind exposure. The potential for dispersal and establishment of tree lichen in regenerated stands with these larger retention patches will probably be much greater compared to the other scenarios. Our modeling approach applying the tree lichen indicator cannot capture the spreading of tree lichen from older into younger stands.

The harvest volumes would increase and remain high in all scenarios. This is to a large part due to the present age-class distribution in the study area, with 70% of the forests younger than 60 years (Fig. 1b). It is, however, unlikely that the trend of increasing harvest volumes would extend much beyond the study period, in any of the scenarios. Economically, the lichen scenarios resulted in 10 to 13% lower NPV compared to the Reference scenario. This is mainly because of more thinnings (both in terms of area and proportion of harvest volume), which are more costly per unit harvested, as well as a lower harvest volume in the future due to lower growth levels. However, the annual net revenues increased in all scenarios over time. This means that the economic return for all scenarios will be higher in the future but on a lower level for the two lichen scenarios. It needs to be stressed that the economic results are limited to wood production, i.e., in this study, we made no attempts to assess the economic value of reindeer husbandry.

The differing snow conditions during winters require a landscape with varying and continuous forests, which offers grazing possibilities at all times (Horstkotte 2013). The results from the scenario analysis can be viewed on a map at different times, thus offering an opportunity to assess landscape patterns. The Ground and tree lichen scenario includes continuous cover forestry and prolonged rotation periods, resulting in more varied forests, with elements of multi-layered canopies, offering more diverse snow and grazing conditions than before. To what extent this improves conditions for grazing dynamics should be explored in future studies.

In the light of the legislatory framework and certification rules, forest companies already today are bound to consider reindeer husbandry. Measures are taken to meet the needs of reindeer husbandry, as reported by the forest companies in consultations informing the analysis. However, our results show that the measures applied in the Reference scenario are not sufficient even to maintain today’s low amount of ground lichen habitat.

Today, many reindeer herders have been forced to switch to supplementary feeding during winter because of limited winter grazing resources. The problem of declining ground lichen resources is further accentuated through negative effects due to climate change and consequent poor and icy snow conditions. Declining lichen resources in combination with difficult snow conditions has led to an unwanted shift away from traditional, natural pasture and natural food-based reindeer husbandry. This constitutes a threat to the persistence of traditional, natural pasture-based reindeer husbandry (Uboni et al. 2020). Hence, a continued “business as usual” forest management reducing the remaining ground lichen habitat even further cannot be seen as an option if the pastoral reindeer husbandry as a basis for the Sami culture is to remain.

Importantly, the adjustments in forest practices in the lichen scenarios do not include new types of silvicultural practices. Instead, adjustments are more related to the timing and intensity of the silvicultural practices that are carried out, which warrants for a relative ease of implementation of the proposed adjustments. In medium to long-term, prescribed burning may have the potential to stop ground lichen decline at landscape scale (Roturier et al. 2023). However, we did not include the effect of prescribed burning as our modelling approach does not allow us to simulate the effects sufficiently well. In addition, there are many practical, regulatory and economic hurdles constraining the implementation of prescribed burning to an extent that would make a difference for lichen availability.

Our results are comparable with those of other studies. Miina et al. (2020) developed a model for predicting ground lichen cover and applied it to evaluate three management scenarios, finding that ground lichen cover decreases in all of them. However, none of the scenarios made specific adaptations to promote lichen habitat. In another study area, Horstkotte et al. (2016) demonstrated a decrease in harvests and revenues of approximately 20% over 100 years when prioritizing reindeer grazing over timber production. Korosuo et al. (2014) found that a continuation of business-as-usual management would continue the decreasing trend in ground lichen area, while implementing continuous cover forestry and precommercial thinnings would halt the decrease and lead to a future increase in the reindeer pasture area to an approximate loss of 5% of net present value for forestry. However, the ground lichen indicator used was less specific, and tree lichen were not included in the study.

Instead of a model predicting the occurrence of ground or tree lichen (Miina et al. 2020), we used simple indicators that are easy to apply in regular forest management planning. The indicators do not predict occurrence, but instead the potential and availability of habitat suitable for lichen occurrence. Thus, our indicators do not account for the time it takes for lichen to establish, nor disappear. For example, exceeding the basal area condition of the ground lichen indicator in a forest stand for only a few years is unlikely to lead to the disappearance of the ground lichen. The advantage of the simplicity of the indicators is that they can be easily used in the forest planning of forest companies or other forest owners, allowing them to assess the impact of their management choices on the lichen potential.

Our results naturally depend on the current state of the forest and can only be generalized to landscapes with similar conditions. However, the chosen indicators and management scenarios are of high relevance also outside our case study area. The lichen indicators and management scenarios have already been used to inform several stakeholder meetings, including meetings organized by the Swedish Forest Agency. We therefore expect that study results can contribute to alleviating the conflict between reindeer husbandry and forestry. This has been possible due to established networks and close contacts with relevant stakeholders throughout the research process. The consultations with RHC and forest companies also helped to identify knowledge gaps and further research needs.

Other positive aspects for biodiversity and recreational values likely accompany adjusting management practices based on the needs of reindeer husbandry. For example, replacing the exotic Lodgepole pine with native Scots pine will benefit biodiversity (Kärvemo et al. 2022). A larger variation in management strategies, including continuous cover forestry and prolonged rotation periods has been shown to benefit multiple forest values including biodiversity and recreation (Eggers et al. 2018, 2019; Eyvindson et al. 2018; Duflot et al. 2021). Likewise, more open forests and forest canopies benefit both ground vegetation diversity (Hedwall et al. 2013) and recreational values. On the other hand, the lower tree growth in the lichen scenarios leads to lower carbon sequestration in living tree biomass compared to the Reference scenario. Future studies should explicitly include indicators for biodiversity and other forest values to study the effects of management aiming at increasing lichen in a wider context.

Climate change is already affecting tree growth (Appiah Mensah et al. 2021), and the impact of a changing climate is expected to increase over time along with rising global temperatures (Lindner et al. 2014). Our analysis accounted for the expected increase in tree growth due to a warmer and longer vegetation period, and an increase in mortality due to disturbances. However, how climate change will play out is highly uncertain, and water limitation may negate the growth-enhancing effect of rising temperatures (Belyazid and Zanchi 2019). Therefore, forest management also needs to focus on promoting resilient forest ecosystems. More research is needed on how climate adaptation can be combined with adaptation to other forest values and uses, including reindeer husbandry and wood production.

## Conclusions

The results of this study can be used to propose and develop a system to support and improve co-planning between reindeer husbandry and forestry with a long time perspective at the landscape scale. We demonstrate a new and improved basis for planning and decision-making on a long-term, which can enable well-informed decisions for a more balanced co-use of the forest. Our results can be used to develop management guidelines to substitute the yearly, stand-based, 3- to 5-year time horizon consultations of today, with an agreement on management guidelines promoting lichen. Applying our proposed system can over time save time for both parties. The heavy burden of the time-consuming consultations have been an issue brought up by RHCs as well as forest companies (Roos et al. 2022). In addition, our results offer a way to put the cards on the table concerning the “costs and benefits” connected to an adapted forest management. Above all, our results can improve conditions for the continuation of the traditional, natural pasture-based reindeer husbandry.

Sami reindeer husbandry is in dire need of improved conditions in winter grazing areas after the last 70 years of declining grazing resources. This is especially critical when also considering increasing pressures from climate change, predation and all other land use forms. Our results show that a continuation of today’s forestry practices would result in further decreases in ground lichen habitat far below today’s already critically low levels. Such declines would constitute a threat to traditional pastoral reindeer husbandry. Tree lichen habitat, on the other hand, can be retained and increased in all scenarios, and may become more important in a changing climate. The forest management strategies proposed to improve conditions for reindeer husbandry present a potential way forward. These strategies result in a 22% increase in ground lichen habitat, with a decrease of 10–13% in net present value from wood production. While the effect on the harvest of sawn timber is relatively minor, pulpwood harvest volume are more affected. Earlier and more intense cleaning and thinning of pine forests to make them more suitable for ground lichen, resulted in a larger volume of pulpwood harvest during the first decades, compared to current practices, and lower pulpwood volumes in the latter half of the study period. In practice, the proposed adapted management strategies are largely part of traditional silvicultural measures, but with changes to the timing and intensity of the measures. Therefore, we assess the technical challenges to implement these adapted strategies to be relatively minor. Our study can provide knowledge where forestry can constitute either a threat to the future of traditional, natural pasture-based reindeer husbandry or where forestry can provide a promising future towards significantly improved conditions for reindeer husbandry.

## Supplementary Information

Below is the link to the electronic supplementary material.

Supplementary file1 (PDF 180 KB)
