# Correction: Adapted forest management to improve the potential for reindeer husbandry in Northern Sweden

**DOI:** 10.1007/s13280-024-02128-y

**Published:** 2025-01-28

**Authors:** Jeannette Eggers, Ulrika Roos, Torgny Lind, Per Sandström

**Affiliations:** Department of Forest Resource Management, Swedish University of Agricultural Sciences, SLU, 901 83 Umeå, Sweden

**Correction to: Ambio 2024, 53:46–62** 10.1007/s13280-023-01903-7

In the original published article, the second sentence in eighth paragraph of “Results” section beginning with “In the Ground lichen and Ground and tree lichen scenarios, NPV was 10% (2669 Eur ha^−1^) and 13% (2880 Eur ha^−1^) lower compared to the Reference scenario, respectively” should have been “In the Ground lichen and Ground and tree lichen scenarios, NPV was 10% (2969 Eur ha^−1^) and 13% (2880 Eur ha^−1^) lower compared to the Reference scenario, respectively”.

The original article has been corrected.

