# Supplementary material for: Adapted forest management to improve the potential for reindeer husbandry in Northern Sweden
Source: Ambio. 2023 Jul 31;53(1):46–62. doi: 10.1007/s13280-023-01903-7 (PMC10692059; doi:10.1007/s13280-023-01903-7)
Supplement: Supplementary file 1 — Supplementary file1 (PDF 180 KB) [file 13280_2023_1903_MOESM1_ESM.pdf]

Ambio

Electronic Supplementary Material

Title: **Adapted forest management to improve the potential for reindeer husbandry in Northern Sweden**

Authors: Jeannette Eggers, Ulrika Roos, Torgny Lind, Per Sandström

Table S1: Percentage lichen cover on pine-dominated[1] productive forest land of total ground layer from the National Forest Inventory (municipalities in the Reindeer Husbandry Area, average 2014-2018)

Site Index, m (Scots pine H_{100})	>50% lichen cover	25-50% cover
10	1	1
11	1	0
12	4	2
13	4	9
14	7	12
15	18	12
16	14	27
17	17	21
18	21	6
19	4	1
20	0	0

[1] Scots pine >65% of basal area, or stem number in young forest <7 m height

Table S2: Percentage of lichen cover (>50% lichen cover, 422 193 ha and 25-50% lichen cover, 572 310 ha) per soil moisture class from the National Forest Inventory (productive forestland, municipalities in the Reindeer Husbandry Area, average 2014-2018).

Soil moisture class	>50% lichen cover	25-50% lichen cover
Dry	33	18
Mesic	66	80
Mesic-moist	2	2
Moist	0	0
Wet	0	0

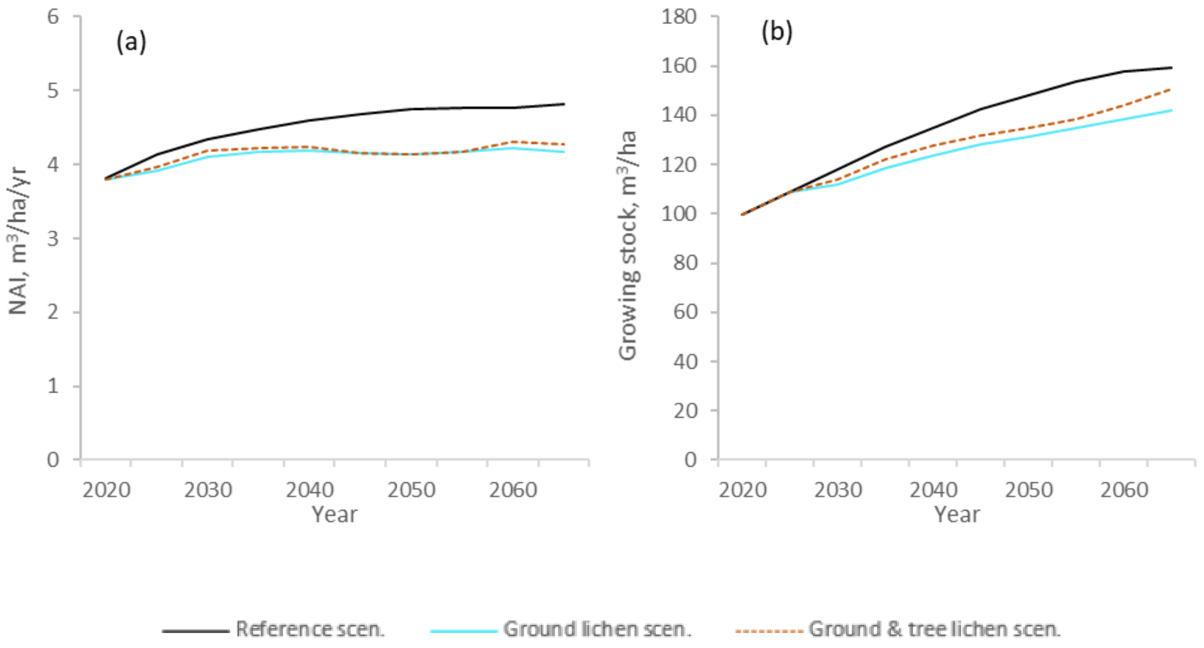

Figure S1. Development of net annual increment (NAI) (a) and growing stock (b) over time.
